# Correction to: What microRNAs could tell us about the human X chromosome

**DOI:** 10.1007/s00018-020-03743-0

**Published:** 2021-01-28

**Authors:** Armando Di Palo, Chiara Siniscalchi, Mariacarolina Salerno, Aniello Russo, Claus Højbjerg Gravholt, Nicoletta Potenza

**Affiliations:** 1 Department of Environmental, Biological and Pharmaceutical Sciences and Technologies, University of Campania “Luigi Vanvitelli”, Caserta, Italy; 2 Pediatric Endocrine Unit, Department of Translational Medical Sciences, University of Naples Federico II, Naples, Italy; 3 Department of Endocrinology and Internal Medicine, Aarhus University Hospital, Aarhus, Denmark; 4 Department of Molecular Medicine, Aarhus University Hospital, Aarhus, Denmark

## Correction to: Cellular and Molecular Life Sciences (2020) 77:4069–4080 10.1007/s00018-020-03526-7

The article What microRNAs could tell us about the human X chromosome, written by Armando Di Palo, Chiara Siniscalchi, Mariacarolina Salerno, Aniello Russo, Claus Højbjerg Gravholt and Nicoletta Potenza as originally published electronically on the publisher’s internet portal (currently SpringerLink) on 30 April 2020 without open access. With the author(s)’ decision to opt for Open Choice the copyright of the article changed on 30 December 2020 to © The Author(s) 2020 and the article is forthwith distributed under the terms of the Creative Commons Attribution 4.0 International License (http://creativecommons.org/licenses/by/4.0/), which permits use, duplication, adaptation, distribution and reproduction in any medium or format, as long as you give appropriate redit to the original author(s) and the source, provide a link to the Creative Commons license and indicate if changes were made.

The original article has been corrected.

